# Are microbes fundamentally different than macroorganisms? Convergence and a possible case for neutral phenotypic evolution in testate amoeba (Amoebozoa: Arcellinida)

**DOI:** 10.1098/rsos.150414

**Published:** 2015-12-16

**Authors:** Angela M. Oliverio, Daniel J. G. Lahr, Jessica Grant, Laura A. Katz

**Affiliations:** 1 Department of Biological Sciences, Smith College, Northampton, MA 01063, USA; 2 Department of Zoology, Institute of Biosciences, University of São Paulo, São Paulo 05508-090, Brazil; 3 Graduate Program in Organismic and Evolutionary Biology, University of Massachusetts, Amherst, MA 01003, USA

**Keywords:** arcellinida, tubulinea, testate amoebae, cryptic species, sex, convergence

## Abstract

This study reveals extensive phenotypic convergence based on the non-monophyly of genera and morphospecies of testate (shelled) amoebae. Using two independent markers, small subunit ribosomal DNA (ssu-rDNA) and mitochondrial cytochrome oxidase I (COI), we demonstrate discordance between morphology and molecules for ‘core *Nebela*’ species (Arcellinida; Amoebozoa). Prior work using just a single locus, ssu-rDNA, also supported the non-monophyly of the genera *Hyalosphenia* and *Nebela* as well as for several morphospecies within these genera. Here, we obtained COI gene sequences of 59 specimens from seven morphospecies and ssu-rDNA gene sequences of 50 specimens from six morphospecies of hyalosphenids. Our analyses corroborate the prior ssu-rDNA findings of morphological convergence in test (shell) morphologies, as COI and ssu-rDNA phylogenies are concordant. Further, the monophyly of morphospecies is rejected using approximately unbiased tests. Given that testate amoebae are used as bioindicators in both palaeoecological and contemporary studies of threatened ecosystems such as bogs and fens, understanding the discordance between morphology and genetics in the hyalosphenids is essential for interpretation of indicator species. Further, while convergence is normally considered the result of natural selection, it is possible that neutrality underlies phenotypic evolution in these microorganisms.

## 1. Introduction

The majority of eukaryotic diversity is microbial, and eukaryotic microbes (i.e. protists) are well recognized as major players in various ecosystem processes (e.g. [1–3]). Our understanding of the molecular diversity across various microbial groups has substantially increased. Yet many microbes exhibit considerable discordance between morphological assessments of biodiversity as compared to molecular estimates. This has presented considerable challenges in untangling the diversification and evolutionary patterns among protists [4]. Thus, the relationship between molecular and morphological diversity in microbes remains a major question in evolutionary biology [5,6]. This is in part owing to uncertainty regarding the relative importance of natural selection and genetic drift in driving phenotypic evolution at the microbial level.

In this study, we use testate (shelled) amoebae as a case study to examine the relationship between morphological and molecular evolution. Lobose testate amoebae (Amoebozoa: Arcellinida) are a clade of microbes that comprise a substantial proportion of biomass in freshwater and terrestrial ecosystems, particularly in peat mosses and soils. Testate amoebae are involved in various ecosystem services [7,8] and may be key players in silica cycling [9–14]. The oldest widely accepted amoebozoan fossils date back to Neoproterozoic (*ca* 750 Ma); these vase-shaped microfossils are interpreted to be Arcellinida (testate amoebae), a view that is corroborated by molecular clocks [4,15]. One particular group of Arcellinida and the focus of this study, the Hyalospheniidae, probably originated in early Carboniferous (approx. 370 Ma) coinciding with widespread colonization of land plants [14]. Understanding the relationship between molecular and morphological diversity in testate amoebae is additionally useful as they are considered biological indicator species [16,17]. Morphospecies have been used to both reconstruct palaeoecological conditions from preserved tests [18–20] and to assess current ecosystem stability and climate changes [21–23].

Testate amoebae are particularly useful to understanding the discordance between molecules and morphology in microbes because they have a long history of studies using microscopy [5]. Testate amoebae are traditionally identified by morphology—namely test size, shape and composition—and the light microscope remains the tool of choice for both palaeontologists and field biologists [24]. Morphological analyses coupled with molecular data have already led to increasing documentation of cryptic species among microbes and within testate amoebae [5,25,26].

Despite this long history, there are significant challenges to uncovering the evolutionary history and phylogenetic relationships of testate amoebae. Within the hyalosphenids (the focus of this study), relatively few taxa have been sampled for molecular data [27,28]. Also, studies reconstructing evolutionary relationships have relied on a single genetic marker to evaluate genetic relationships (either nuclear small subunit ribosomal DNA (ssu-rDNA) or mitochondrial cytochrome oxidase I (COI)). The small subunit rDNA gene is not variable enough to resolve relationships within hyalosphenids, though it provides considerable power at deeper levels [25,27,29]. Mitochondrial COI is often used for DNA barcoding species, where the main goal is to identify an unknown sample, and has demonstrated utility within hyalosphenids for cryptic species delineation [25,26,30]. However, any single marker to determine evolutionary relationships remains controversial [31–33]. Will *et al*. [33] advocate for an ‘integrative taxonomy’ including multiple markers and other types of data (morphological and ecological) to identify taxa at all systematic levels. Such integrative taxonomy is difficult in testate amoebae: these taxa have proved challenging for traditional polymerase chain reaction (PCR) methods [6] and they are not good candidates for high-throughput sequencing (species are currently uncultivable, sometimes grow with algal symbionts, and are often covered with other microbes such as fungi and choanoflagellates).

Here, we consider evolution of test morphology among hyalosphenids, including morphospecies within the genera *Nebela*, *Quadrulella* and *Hyalosphenia*. We compare insights from two independent markers, mitochondrial COI and nuclear ssu-rDNA, to increase power compared to previous single locus studies. Our goals are: (i) to refine our understanding of the diversity that exists within the hyalosphenids by comparing ssu-rDNA, a well-conserved marker, with COI, a fast-evolving marker across specimens, and (ii) to elucidate the evolutionary patterns in changing test morphology by exploring multiple hypotheses, including assessing the possibility of convergence or parallelism among neutral phenotypes.

## 2. Results

### 2.1 Characterization of morphospecies

**Figure 1.**
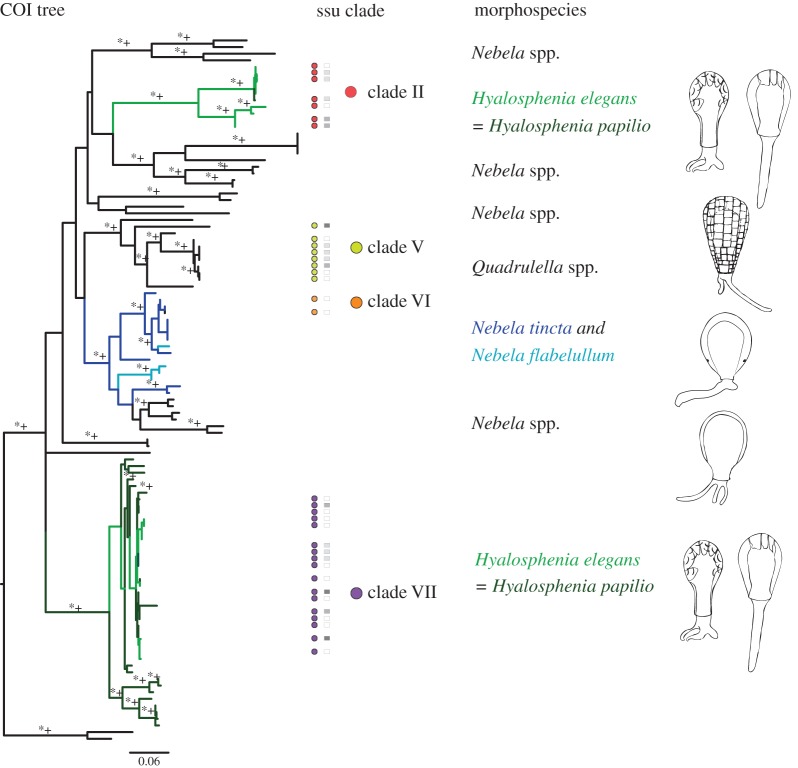
Relationships among the ‘core *Nebelas*’ highlighting morphological traits. Tree based on COI ML reconstructions (a highly similar topology was derived from Bayesian Inference using MrBayes). Branches where morphospecies are monophyletic are coloured black. Branches where morphospecies are not monophyletic are coloured corresponding to the morphospecies. Coloured circles are overlain where we have obtained the corresponding ssu-rDNA for the specimen. Circle colour signifies clade location of specimen on ssu-rDNA ML tree (see key). Corresponding sub clades are overlain as squares. Asterisks indicate bootstrap support is 70% or greater and plus signs indicate posterior probability support is 0.70 or greater. The scale bar represents 0.06% sequence divergence.

**Figure 2.**
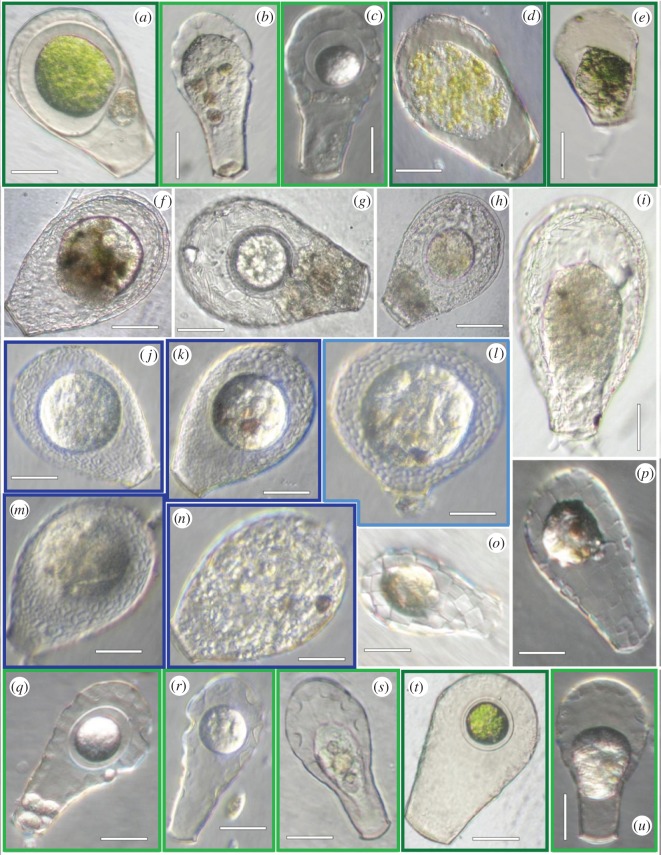
Light images of organisms used in this study, organized by clade (table 1). (*a*–*e*) *Hyalosphenia papilio* and *H. elegans* from clade 7. (*f*–*i*) *Nebela carinata* and *N. marginata* from clade 3. (*j*–*n*) *Nebela tincta* and *N. flabelullum* from clade 6. (*o*,*p*) *Quadrulella* from clade 5, Quad. (*q*–*u*) *Hyalosphenia* *papilio* and *H. elegans* from clade 2. Frame colours correspond to non-monophyletic morphospecies in figure 1.

Our analyses are based on an overlapping sample of COI and ssu-rDNA sequences from seven morphospecies within the ‘core *Nebelas*’. We obtained partial COI gene sequences of 59 specimens from seven morphospecies: *Hyalosphenia elegans* (*n*=19), *Hyalosphenia papilio* (*n*=17), *Nebela carinata* (*n*=3), *Nebela marginata* (*n*=2), *Nebela tincta* (*n*=8), *Nebela flabelullum* (*n*=1) and *Quadrulella symmetrica* (*n*=9; electronic supplementary material, table S2). Alignment of the COI locus was 669 bp and included 106 taxa (from this study and GenBank; electronic supplementary material, tables S1 and S2, and figure S1a,b; figure 1). All specimens analysed were first characterized by light microscopy (figure 2; [7,34,35]). Alignment of the COI sequences revealed clade-specific, single nucleotide deletions. This suggests that there may be RNA editing of these mitochondrial genes: at seven different points along the COI alignment, there were ‘missing’ nucleotides in some of the sequences (electronic supplementary material, table S3). These gaps were consistent across clades (either all sequences in a clade had a gap at a given location or none did). We infer by comparisons among species that the ‘missing’ nucleotides are generally C or T, consistent with data from mitochondria of other Amoebozoa [36–38].

In addition to our COI data, we obtained partial ssu-rDNA gene sequences of 50 specimens from six morphospecies of ‘core *Nebelas*’ within Arcellinida: *H. elegans* (*n*=12), *H. papilio* (*n*=14), *N. carinata* (*n*=1), *Nebela tubulosa*(*n*=3), *N. tincta* (*n*=2) and *Q. symmetrica* (*n*=18; electronic supplementary material, table S2). Alignment of the ssu-rDNA locus was 507 bp and 123 taxa were included (electronic supplementary material, tables S1 and S2, and figure S1). We also incorporated ssu-rDNA sequences of previously sequenced specimens for which we generated COI data (table 1).

**Table 1.** Number of observed specimens in ‘core *Nebela*’ clades for both COI and ssu-rDNA.

genus	COI	small subunit	no. overlap^a^	light images (figure 2)	clade reference no.’s (electronic supplementary material, figure S1)
*Nebela*	4	^b^			1
*Hyalosphenia*	10	18	7	*q*–*u*	2
*Nebela*	9	11^b^		*f*–*i*	3
*Nebela*	4	5			4
*Quadrulella*	11	21	8	*o*,*p*	5
*Nebela*	22	14	2	*j*–*n*	6
*Hyalosphenia*	41	46	17	*a*–*e*	7

^a^‘no. overlap’ refers to cells in which both COI and ssu-rDNA data were obtained.

^b^Signifies an unresolved clade in ssu-rDNA reconstruction and/or no representative taxa.

### 2.2 Phylogenetic reconstruction of cytochrome oxidase I data and approximately unbiased test for monophyly

Phylogenetic reconstructions based on COI data highlight multiple instances where relationships among genera and species are inconsistent with morphology (figure 1). Taxa fall within seven major clades, using *Padaungiella nebeloides* and *Padaungiella lageniformis* as outgroups following analysis by Kosakyan *et al*. [26]. Trees inferred with maximum-likelihood (ML) and Bayesian approaches have very similar topologies with no conflict among supported nodes, so we present only the ML tree with Bayesian posterior probabilities mapped on key nodes. We focus on four of the seven major clades, which indicate the non-monophyly of the genus *Hyalosphenia*, and the non-monophyly of nominal taxa (i.e. morphospecies) *H. elegans*, *H. papilio*, *N. tincta* and *N. flabelullum*.

In our COI tree, *H. elegans* and *H. papilio* fall within two distinct clades with full support from both ML and Bayesian analyses (99% *b*oot*s*trap (BS)/1.00 *p*osterior *p*robability (PP) for both clades; figure 1 and electronic supplementary material, figure S1). In both (clade II and clade VII), *Hyalosphenia* morphospecies are interdigitated. *Hyalosphenia elegans* and *H. papilio* have three instances of identical sequences: (i) *H. papilio* KP691368 and *H. elegans* KP691367; (ii) *H. papilio* KP691389 and *H. elegans* KP691380, KP691381 and KP691382; and (iii) *H. papilio* KP691373 and *H. elegans* KP691374. The two *Hyalosphenia* clades are considerably divergent with up to 23% divergence between clades. There is also significant diversity among morphospecies within both clades. In clade VII (figure 1), there are 28 distinct haplotypes of *H. papilio* and six haplotypes of *H. elegans*, with divergences within morphospecies of up to 18.7% and 18.4%, respectively. The other *Hyalosphenia* clade (clade II, figure 1) contains four distinct haplotypes of *H. elegans* and one haplotype of *H. papilio* with divergence up to 12.4% among *H. elegans*.

Other instances of non-monophyly are found in moderately supported clade VI (85% BS/0.56 PP; figure 1). Clade VI includes *N. tincta*, *N. guttata*, *N. flabelullum*, *N. pechorensis*, *N. rotunda* and *N. collaris*. The morphospecies *N. tincta* and *N. flabelullum* are not monophyletic and *N. flabelullum* clusters within *N. tincta* in two spots: *N. flabelullum* KP691357 with *N. tincta* var. major JN849067.1 (55% BS/1.0 PP) and three *N. flabelullum* (JN849026.1, KJ544158 and KJ544158) between two subclades containing *N. tincta* (100%/—BS). Additionally, *Nebela speciosa* (JN849045) and two *N. tincta* (KP691349 and KP691350) may be non-monophyletic as they are unresolved in this reconstruction. Other clades are made up of monophyletic *Nebela* species including clade I (99% BS/1.00 PP), clade III (71% BS/0.84 PP), clade IV (—BS/0.99PP) and clade V (79 BS/0.68 PP).

To determine with certainty that the monophyly of apparently non-monophyletic taxa could be rejected (figure 1, coloured branches), we ran an approximately unbiased test (AU; table 2). The AU test results reject the monophyly of morphospecies *H. elegans* (*p*=2×10^−7^), *H. papilio* (*p*=7×10^−36^), *N. tincta* (*p*=3×10^−47^) and *N. flabelullum* (6×10^−58^). AU tests also reject the monophyly of genera *Hyalosphenia* and *Nebela* (table 1, *p*=2×10^−64^ and *p*=4×10^−93^, respectively).

**Table 2.** AU test results of genera and morphospecies. (The constraint-tested column refers to proposed taxonomic groups that were constrained to test for monophyly through the AU; weighted Kishino–Hasegawa test (WKH); and the weighted Shimodaira–Hasegawa test (WSH). Bold results indicate relationships that we reject as monophyletic. COI ML tree (electronic supplementary material, figure S1) used for AU tests.)

constraint tested	AU	WKH	WSH
*Hyalosphenia*	**2**×**10**^−**64**^	**0**	**0**
*Nebela*	**4**×**10**^−**93**^	**0**	**0**
*H. elegans*	**2**×**10**^−**7**^	**0**	**0**
*H. papilio*	**7**×**10**^−**36**^	**0**	**0**
*N. flabelullum*	**6**×**10**^−**58**^	**0**	**0**
*N. tincta*	**3**×**10**^−**47**^	**0**	**0**

### 2.3 Concordance of mitochondrial and nuclear sequences across major clades

We characterized both ssu-rDNA and COI data for 34 specimens (indicated by circles that refer to the SSU sequences in figure 1). These comparisons between the mitochondrial COI data and the independently evolving nuclear ssu-rDNA reveal concordance across major clades (figure 1). Mapping the ssu-rDNA clades onto the COI tree (figure 1, coloured branches) supports two distinct and divergent *Hyalosphenia* clades for both genes. Other clades are also consistent across both genetic markers, such as the *Quadrulella* clade and clade containing *N. tincta* and *N. flabelullum* (figure 1). The full COI and ssu-rDNA gene trees with support values are found in the electronic supplementary material, figure S1.

### 2.4 Biogeography and divergence within clades

Based on our sampling, there is some evidence of clustering of taxa by collection location (table 1). For instance, all *H.* *papilio* from Europe sampled in Kosakyan *et al*. [26] form a monophyletic group in clade VII, and only one clade of *N. carinata* was found by Kosakyan *et al*. [26]. *Hyalosphenia papilio* from Canada and Switzerland sampled in Fiz-Palacios *et al*. [4] also form a unique monophyletic group in clade VII. Further, the group in clade II that contains *H. elegans* and *H. papilio* was found only in this study. This may reflect a specificity of clade II to certain sampling locations. However, some haplotypes were shared across various sampling locations, for instance one haplotype of *N. marginata* was recovered from both Massachusetts (this study) and Europe [26].

## 3. Discussion

The two main findings from our analysis of hyalosphenids within Arcellinida are that: (i) some specimens of *H. papilio* share identical sequences of both COI and ssu-rDNA with *H. elegans*, and (ii) there may be convergent evolution of test morphology across the two distinct, non-monophyletic *Hyalosphenia* clades (figure 1). These data raise questions on the evolutionary mechanisms driving morphology at the microbial scale.

### 3.1 Interdigitation of *Hyalosphenia elegans* and *Hyalosphenia papilio* within a given *Hyalosphenia* clade

We confirm identical sequences among *H.* *elegans* and *H. papilio* with a second marker, COI (table 1), as previously observed only in the more conserved ssu-rDNA [29]. There are both interdigitation and identical sequences shared between the morphospecies *H. papilio* and *H. elegans*. This raises interesting questions about what biological processes might be involved. We will discuss several alternative hypotheses that might explain the genetic patterns we observed, and briefly suggest some future tests to elucidate what biological processes are occurring.

The interdigitation of morphospecies *H. elegans* and *H. papilio* within a clade may be attributed to: (i) incomplete lineage sorting, (ii) alternate stages in a life cycle, or (iii) the presence of two mating types within a single species. The first formal possibility is that the interdigitation *of H. elegans* and *H. papilio* is owing to incomplete lineage sorting (ancestral polymorphisms shared by two now-isolated species). However, incomplete lineage sorting is more likely for many closely related specimens (i.e. short branches within clades) and thus seems less likely to occur across two disparate clades. An alternative hypothesis is that *H. elegans* and *H. papilio* are two different stages within a life cycle, as we discussed in Oliverio *et al*. [29]. This may be seasonally driven. *Hyalosphenia papilio* and *H. elegans* are, respectively, more abundant in spring to summer and summer to autumn [39] suggesting the possibility of two seasonal morphotypes. Observing such a transition between life stages or perhaps an intermediate form would lend great support to this idea, but such experiments are challenging given that these testate amoebae are not yet cultivable.

Another possibility is that the two morphospecies are two mating types within a single species, as we first propose in Oliverio *et al*. [29]. While testate amoebae are generally believed to be asexual, recent literature suggests this may not be the case—as described in reviews of sex in eukaryotes [40,41]. We also previously reported two possible sightings of mating between *H. papilio* and *H. elegans* ([29] see: http://www.science.smith.edu/departments/Biology/lkatz/movies/TestateAmoebae.html). Based on these observations, it may be the case that *H. elegans* and *H. papilio* represent two different mating types of the same species. Ultimately, sequencing larger portions of the nuclear genome will allow for the assessment of introgression between these morphospecies.

### 3.2 Non-monophyly *Hyalosphenia* genus suggests convergence or parallel evolution of test shape

Mitochondrial COI data confirm discordance between morphology and genetics at the species and genus level within the ‘core *Nebela*’ as first observed with ssu-rDNA [27,29]. These results are consistent with two prior studies, Lara *et al*. [27] and Oliverio *et al*. [29]. Here, two non-monophyletic clades of *Hyalosphenia*were also recovered using ssu-rDNA. By contrast, two other phylogenetic studies of the ‘core *Nebelas*’ [4,26] using the COI marker only recover one of the major *Hyalosphenia* clades. It is possible that the recovery of only one *Hyalosphenia*clade in these COI studies may be a result of limited sampling (e.g. *H. elegans* was not included in either study).

Other morphospecies in the ‘core *Nebela*’ are also morphologically and genetically discordant in our COI reconstruction, consistent with patterns first observed in ssu-rDNA [27,29]. For example, *N. tincta* and *N. flabelullum* are also interdigitated with both markers; however sequences are never identical (figure 1). This could be owing to low sampling (for *N. flabelullum* *N*=2 in COI data), misidentification (there are many cryptic species within the *N. tincta–bohemica–collaris* [25] or incomplete lineage sorting and/or recombination (as previously discussed).

The non-monophyly of genera indicates remarkable phenotypic convergence or parallel evolution, as the morphologies that underlie the genera *Nebela* and *Hyalosphenia* have evolved multiple times (figure 1). We discuss possible implications and mechanisms for both convergence and parallel evolution. One possibility is that the retention of conserved test morphology is owing to parallel evolution such that distantly related lineages retain ancestral phentoypes. Such parallel evolution probably explains the similarity in the biflagellate spores found in distantly related stramenopile lineages such as labyrinthulids and oomycetes.

An alternative hypothesis is that the two *Hyalosphenia* clades are a result of extensive phenotypic convergence. While we see both convergence or parallelism as being plausible, the explanations of parallelism are less parsimonious than convergence given our tree. Parallelism requires eight evolutionary steps, whereas convergence requires two. There are many examples of phenotypic convergence in microbial lineages [5,42,43]. Such convergence may be owing to either selection or neutral processes (reviewed in Lahr *et al*. [5]). Convergence *via* selection has been well documented, particularly in macroorganisms [42,44,45]. For example, a study of open-habitat chats (genera *Myrmecocichla*, *Cercomela*, *Oenathe*) found non-monophyly across all genera and suggest that there is extensive convergence of plumage patterns of distantly species of these birds [46]. Many examples of selective convergence also come from plants (e.g. [44]). Other studies have suggested that a strong selection on body morphology confounds our understanding of evolutionary relationships [47,48]. Thus, it is possible that morphology in the ‘core *Nebelas*’ evolved multiple times from some sort of environmental or ecological pressures. For example, maybe the clear (i.e. hyaline) test of *H. papilio* (figure 2) converged due to selection for harbouring photosynthetic symbionts.

An alternative explanation for convergent evolution is that the similar test forms in *Hyalosphenia* and *Nebela* are neutral and appear multiple times due to chance. In *Randomness and Evolution*, John Tyler Bonner argues that morphological differences in smaller organisms are often more likely to be the result of stochastic processes rather than selection [49]. Bonner suggests that because morphological space is constrained (e.g. there are only a limited number of possible body plans) and microbial-scale morphologies are under less selection, convergent morphologies can arise due to chance. Because of the possible influence of neutrality of phenotypes at the microbial level, we suspect Bonner’s conjecture. That discordance between morphology and molecules may be more neutral than selective in microbes may be the case within testate amoebae in particular [49]. Lahr *et al* [5] discuss the challenges in testing hypotheses of neutrality as a driving force in phenotypes at the microbial level, though methodological advancements may make such tests easier once genome scale data emerge from these lineages. Moreover, acknowledging the discordance between morphology and genetics in microbes such as the testate amoebae discussed here is essential for understanding fundamental questions on both the ecological roles and evolutionary forces that drive the evolution of testate amoebae.

## 4. Material and methods

### 4.1 Study sites and field methods

Samples for this study were collected between 2009 and 2013 from Hawley Bog Preserve, Charlemont, MA (N 42.583, W −72.883), Bear Swamp, Ashfield, MA (N 42.535 and W −72.830) and Big Heath, Acadia National Park, ME (N 44.233, W −68.319). At each site, approximately 4 cm^3^ of surface moss was collected from several different sampling locations. *Sphagnum* samples were stored in a light incubator until use.

### 4.2 DNA isolation and documentation, polymerase chain reaction amplification and sequencing

Amoebae were isolated from *Sphagnum* samples via filtering through a 3 cm sieve and then added to autoclaved bog/fen water collected on site. Genetic material was acquired through three methods: (i) genomic amplification, (ii) phenol/chloroform extraction, and (iii) cDNA construction. For all methods, amoebae were washed once in filtered, autoclaved water from the sample location, left overnight to digest prey and then washed twice more in autoclaved water to eliminate possible contamination. During isolation, we photodocumented specimens with a Canon® Powershot A640 digital Camera on a Nikon® Eclipse TS100 inverted microscope. Images were reviewed in Adobe® Photoshop CS5. Some specimens were isolated and identified before the taxonomic actions proposed by Kosakyan *et al*. [25,26] and we do not believe we can adequately discriminate into the different proposed species (particularly in the *N. tincta–bohemica–collaris* complex) based on our images. Thus, we note our specimens labelled *N. tincta* may belong to several different species as defined by Kosakyan *et al*. [25].

For single-cell genomic amplification, single cells were then placed in DLB buffer and amplified with Repli-g UltraFast Mini Kit (Qiagen, Cat. No. 150025) following manufacturer’s protocols. For phenol/choloroform extraction, approximately 50 cells were placed in DNA prep buffer and extracted following phenol/choloroform protocol [50]. For cDNA construction, five to 10 cells were placed in resuspension buffer with lysis enhancer from a SuperScript III CellsDirect cDNA synthesis kit (Invitrogen, Cat. No. 18080-200) and reverse transcribed from RNA to cDNA following manufacturer’s directions. For cDNA and phenol/chloroform, 25–50 cells were picked from one sample by morphotype identification based on light microscopy. There may be morphological differences among cells picked and pooled. However, our goal here was to confirm that we find the same haplotypes as with single-cell amplification.

PCRs were performed with Phusion HotStart polymerase (New England BioLabs, Cat. No. F540) according to standard protocols, using ‘core *Nebelas*’ specific primers for both ssu-rDNA and COI reactions. For ssu-rDNA, several different primers were used. The first method was used on all cells prepared before August 2012 and the second method was used on all cells prepared on August 2012 or later. For the first method, a PCR reaction was performed using the primers Hyalo 520+5^′^-(GGGYCACTTGTATTGAGCCGTC)-3^′^ and Hyalo 1648−5^′^-(CTYMGGGYAAGGGWKATTGC)-3^′^. For the second method, two nested PCR reactions were performed when using the primers HYI+5^′^-(ATCAAGAACRAAAGTTGCRGGCGC)-3^′^, HYI- 5^′^ (TGYRGCCCAGAACATCTAAGGGC)-3^′^ and then HYO+5^′^-(GGACCRGTTCAAGACAAACTASTGC)-3^′^, HYO- 5^′^-(GAGCATKTCATTGYAACGCGC)-3^′^ with a 1 : 50 dilution between reactions. For COI PCRs, we used LCO1490 and HCO2198 for the first PCR reaction [51] and then ArcelCOIF a primer designed to amplify ‘core *Nebelas*’ [26] for the second reaction along with HCO2198. Again, there was a 1 : 50 dilution between reactions.

Genetic material was serially diluted from 1 to 1 : 100 in preparation for PCR. Negative controls were used in all reactions. PCR conditions were optimized for each primer set. PCR products were gel-isolated using the Cleanspin Gel DNA Recovery Kit (The Gel Company, San Francisco, Cat No. DRK-200) and cleaned either by the Microclean kit (The Gel Company) or by USB® ExoSAP-IT® PCR product clean up (Affymetrix). Zero Blunt TOPO PCR Cloning Kit (Invitrogen, Cat. No. K280020) was used following the manufacturer’s protocols for cloning. Eight clones were picked for each isolate. AmpliTaq-gold polymerase and vector primers (Applied Biosystems) were used to screen clones. Sequencing reactions were prepped using a Big Dye Terminator v. 3.1 Cycle sequencing Kit (Applied Biosystems) and run at the Center for Molecular Biology (Smith College, Northampton, MA) on an ABI 3100 automated sequencer.

### 4.3 Analytical methods

For both ssu-rDNA and COI, sequences were examined with SeqMan v. 6.1 (DNASTAR, Inc., Madison, WI, USA). Regional similarities between the sequences were compared using Basic Local Alignment Search Tools. More sequences of taxa in *Hyalosphenia* and *Nebela* were added from GenBank to both alignments as well as outgroups. Sequence alignments were constructed as per Oliverio *et al*. [29].

Phylogenetic reconstructions were made using ML and Bayesian methods for ssu-rDNA and COI. For COI, the ML tree was built using the RAxML-HPC2 on XSEDE v. 7.6.3 [52] via the CIPRES Science Gateway Portal v. 3.3 [53], using the GTR+I+G model with 100 bootstrap replicates. We included *P. lageniformis*, *P. wailesi* and *P. nebeloides* to root all trees as per the phylogeny in Kosakyan *et al*. [26]. The best-scoring ML tree was compared to one acquired through Bayesian analysis, using MrBayes v. 3.1.2 [54] through the online server CIPRES Portal [53], and a highly similar topology was obtained. Two simultaneous Markov chain Monte Carlo chains, with 1 000 000 generations were used. For every 1000th generation, the best likelihood tree was selected. The burn in was set at 25%. The consensus tree was built using the 50% majority-rule, and posterior probabilities were calculated for each node. Distances among taxa and across clades were measured using distance estimation in MEGA v. 4.0 software [55] with a Maximum Composite Likelihood model with a gamma distribution. Gaps and missing data were dealt with by pairwise deletion and uncorrected distances were used.

For ssu-rDNA, the ML tree was built as per Oliverio *et al*. [29] with PhyML v. 3.0 (2012) software [56] using the GTR-I model of nucleotide substitution (electronic supplementary material, figure S2). The taxa *Apodera vas* and *P. lageniformis* were included as outgroups for the analyses.

The AU test [57] was used to test the monophyly of several proposed genera and species in the COI alignment (figure 1 and electronic supplementary material, figure S1). The AU test evaluates the per-site likelihoods of unconstrained versus constrained trees, to assess whether we can reject the monophyly of constrained clades given the available data. Here, we created constraints and generated corresponding ML reconstructions and per-site likelihoods for six hypotheses that were not monophyletic in the best-scoring ML tree, including two genera and four morphospecies (table 2). Constraint tree searching parameters were identical to the best-scoring unconstrained reconstruction. The per-site likelihoods of constrained trees were all compared to those of the best unconstrained tree using CONSEL software [58] with standard parameters to find all statistical outputs.

## Supplementary Material

Cox1_suppdata_resub.pdf

## Supplementary Material

cox1_suppfigs.pdf
