# Supplementary material for: Are microbes fundamentally different than macroorganisms? Convergence and a possible case for neutral phenotypic evolution in testate amoeba (Amoebozoa: Arcellinida)
Source: R Soc Open Sci. 2015 Dec 16;2(12):150414. doi: 10.1098/rsos.150414 (PMC4807447; doi:10.1098/rsos.150414)
Supplement: Cox1_suppdata_resub.pdf [file rsos150414supp1.pdf]

Supplementary data

Table S1. List of all taxa in COI and 18S rDNA reconstructions from Genbank.
Table S2. List of all taxa from this study used in COI and 18S rDNA reconstructions with corresponding field and prep data.
Table S3. Inferred nucleotides from gaps in the COI alignment.

Figure S1. Phylogenies for both genes surveyed in the present study that were overlayed in Figure 1. **a.** COI tree of 'core Nebelas' obtained by maximum likelihood analysis using PhyML. Numbers at nodes are bootstrap values, shown support is 50% or greater. **b.** 18S rDNA tree of 'core Nebelas'. This tree was obtained by maximum likelihood analysis using PhyML, and numbers at the nodes are statistical support expressed as SH-like aLRT probabilities.

Table S1. List of all taxa from GenBank included in this study.

SSU		COI	
Taxon	Genbank ID	Taxon	Genbank ID
Apodera vas	EU392156	*H. papilio*	JN849014.1
H. elegans	EU392154	*H. papilio*	JN849018.1
H. elegans	KF476065	*H. papilio*	JN849019.1
H. elegans	KF476066	*N. tubulosa*	JN849022.1
H. elegans	KF476071	*N. tincta var galeata*	JN849023.1
H. elegans	KF476077	*N. tincta*	JN849025.1
H. elegans	KF476078	*N. flabellulum*	JN849026.1
H. elegans	KF476091	*N. marginata*	JN849031.1
H. elegans	KF476104	*N. carinata*	JN849036.1
H. elegans	KF476105	*N. carinata*	JN849041.1
H. elegans	KF476106	*N. speciosa*	JN849045.1
H. elegans	KF476107	*Q. symmetrica*	JN849048.1
H. elegans	KF476109	*Q. longicollis*	JN849050.1
H. papilio	AY848966	*N. AK-2011N_meis62*	JN849052.1
H. papilio	EU392153	*N. ansata*	JN849055.1
H. papilio	JF694282	*N. hippocrepis*	JN849057.1
H. papilio	KF476064	*N. galeata*	JN849058.1
H. papilio	KF476068	*N. tubulosa*	JN849061.1
H. papilio	KF476072	*N. penardiana*	JN849062.1
H. papilio	KF476073	*N. nebeloides*	JN849063.1
H. papilio	KF476074	*N. (P.) lageniformis*	JN849065.1
H. papilio	KF476075	*N. tincta major*	JN849067.1
H. papilio	KF476076	*N. pechorensis*	JX682577.1
H. papilio	KF476079	*N. pechorensis*	JX682583.1
H. papilio	KF476080	*N. collaris*	JX682586.1
H. papilio	KF476081	*N. rotunda*	JX682591.1
H. papilio	KF476083	*N. rotunda*	JX682595.1
H. papilio	KF476084	*N. tincta*	JX682597.1
H. papilio	KF476086	*N. guttata*	JX682598.1
H. papilio	KF476087	*N. tincta*	JX682599.1
H. papilio	KF476090	*H. papilio*	KJ544147.1
H. papilio	KF476093	*H. papilio*	KJ544148.1
H. papilio	KF476094	*H. papilio*	KJ544151.1
H. papilio	KF476095	*H. papilio*	KJ544152.1
H. papilio	KF476096	*H. papilio*	KJ544153.1
H. papilio	KF476097	*H. papilio*	KJ544155.1
H. papilio	KF476098	*N. collaris*	KJ544161.1

H. papilio	KF476102	*N. flabellulum*	KJ544158.1
H. papilio	KF476103	*N. flabellulum*	KJ544159.1
N. (P.) lageniformis	EU392155.1	*N. marginata*	KJ544160.1
N. ansata	HMO50411	*N. sp. TJT-2014*	KJ544164.1
N. ansata	HMO50412	*H. papilio*	KC170421.1
N. ansata	HMO50413	*H. papilio*	KC170429.1
N. carinata	EU392143	*H. papilio*	KC170441.1
N. carinata	EU392144	*H. papilio*	KC170453.1
N. carinata	JF694283	*H. papilio*	KC170582.1
N. carinata	KF476101	*H. papilio*	KC170583.1
N. flabellulum	EU392152		
N. flabellulum	KF476112		
N. flabellulum	KF476113		
N. flabellulum	KF476114		
N. militaris	KF476116		
N. militaris	KF476118		
N. militaris	KF476119		
N. militaris	KF476120		
N. militaris	KF476121		
N. militaris	KF476123		
N. militaris	KF476129		
N. penardiana	EU392145		
N. penardiana	EU392146		
N. tincta	AY848968		
N. tincta	EU392149		
N. tincta	EU392150		
N. tincta	EU392151		
N. tincta	KF476099		
N. tincta	KF476125		
N. tincta	KF476127		
N. tubulosa	EU392148		
N. tubulosa	KF476117		
N. tubulosa	KF476122		
Q. symmetrica	KF476100		
Q. symmetrica	KF476110		
Q. symmetrica	KF476111		

Table S2. List of all taxa in reconstructions including sequences from this study.

Taxon	SSU Genbank ID	COI Genbank ID	Site	Collection date	Prep date	# days in culture	Cell prep ID
H. elegans	KR063292	KP691377	Hawley Bog	10/7/12	4/9/13	184	HE_AO_4.100.18
H. elegans	KR063293	KP691380	Hawley Bog	10/7/12	4/9/13	184	HE_AO_4.100.20
H. elegans	KR063294	KP691378	Hawley Bog	10/7/12	4/9/13	184	HE_AO_4.100.22
H. elegans	KR063295	KP691381	Hawley Bog	10/7/12	4/9/13	184	HE_AO_4.100.24
H. elegans	KR063296	KP691382	Hawley Bog	10/7/12	4/9/13	184	HE_AO_4.100.26
H. elegans	KR063297	KP691340	Hawley Bog	4/29/13	4/30/13	1	HE_AO_4.117.3
H. elegans	KR063298	KP691341	Hawley Bog	4/29/13	4/30/13	1	HE_AO_4.117.7
H. elegans	KR063299	KP691343	Hawley Bog	4/29/13	5/3/13	4	HE_AO_4.125.11
H. elegans	KR063300	KP691342	Hawley Bog	4/29/13	5/3/13	4	HE_AO_4.125.9
H. papilio	KR063301	KP691387	Hawley Bog	3/23/12	4/1/12	9	HP_AO_2.08.10
H. papilio	KR063302	KP691389	Hawley Bog	3/23/12	4/1/12	9	HP_AO_2.08.7
H. papilio	KR063303	KP691388	Hawley Bog	3/23/12	4/1/12	9	HP_AO_2.08.8
H. papilio	KR063304	KP691373	Hawley Bog	10/7/12	4/9/13	184	HP_AO_4.100.15
H. papilio	KR063305	KP691339	Acadia	8/10/12	2/15/13	189	HP_AO_4.52.9
H. papilio	KR063306	KP691383	Hawley Bog	4/7/10	4/9/10	2	HP_DL3.99.2
N. carinata	KR063307	KP691346	Acadia	8/10/12	2/15/13	189	NC_AO_4.52.6
N. tincta	KR063308	KP691351	Acadia	8/10/12	11/6/12	88	NT_AO_2.21.6_3
Q. symmetrica	KR063309	KP691358	Bear Swamp	10/11/11	11/7/11	27	QS_AO_1.66.1
Q. symmetrica	KR063310	KP691366	Bear Swamp	10/11/11	12/6/11	56	QS_AO_1.80.2
Q. symmetrica	KR063311	KP691365	Bear Swamp	10/11/11	12/6/11	56	QS_AO_1.80.3
Q. symmetrica	KR063312	KP691364	Bear Swamp	10/11/11	12/6/11	56	QS_AO_1.80.4
Q. symmetrica	KR063313	KP691362	Bear Swamp	10/7/12	2/13/13	129	QS_AO_4.48.18
Q. symmetrica	KR063314	KP691361	Bear Swamp	10/7/12	3/26/13	170	QS_AO_4.79.6
Q. symmetrica	KR063315	KP691360	Bear Swamp	10/7/12	3/26/13	170	QS_AO_4.79.7
H. elegans	KR063316	n/a	Acadia	8/10/12	8/10/12	0	HE_ANP1
H. elegans	KR063317	n/a	Hawley Bog	10/11/11	3/4/12	145	HE_AO_1.122.23

H. elegans	KR063318	n/a	Hawley Bog	10/11/11	11/8/11	28	HE_AO_1.67.6
H. papilio	KR063319	n/a	Acadia	8/10/12	8/10/12	0	HP_ANP_3wo.10
H. papilio	KR063320	n/a	Acadia	8/10/12	8/10/12	0	HP_ANP_3wo.3
H. papilio	KR063321	n/a	Hawley Bog	3/23/12	4/1/12	9	HP_AO_2.08.1
H. papilio	KR063322	n/a	Hawley Bog	3/23/12	4/1/12	9	HP_AO_2.08.4
H. papilio	KR063323	n/a	Acadia	8/10/12	9/19/12	40	HP_AO_2.65.1
H. papilio	KR063324	n/a	Acadia	8/10/12	9/19/12	40	HP_AO_2.65.12
H. papilio	KR063325	n/a	Hawley Bog	10/7/12	4/9/13	184	HP_AO_4.100.7
H. papilio	KR063326	n/a	Hawley Bog	4/7/10	4/9/10	2	HP_DL3.99.3.1.1
N. tincta	KR063327	n/a	Acadia	8/10/12	11/5/12	87	NT_AO_2.117.2
N. tubulosa	KR063328	n/a	Hawley Bog	10/7/12	2/13/13	129	NTU_AO_4.49.2
N. tubulosa	KR063329	n/a	Acadia	8/10/12	2/15/13	189	NTU_AO_4.52.11
N. tubulosa	KR063330	n/a	Acadia	8/10/12	3/26/13	228	NTU_AO_4.79
Q. symmetrica	KR063331	n/a	Bear Swamp	10/11/11	11/7/11	27	QS_AO_1.66.3
Q. symmetrica	KR063332	n/a	Bear Swamp	10/11/11	11/7/11	27	QS_AO_1.66.4
Q. symmetrica	KR063333	n/a	Bear Swamp	10/7/12	1/5/13	90	QS_AO_4.36.4
Q. symmetrica	KR063334	n/a	Bear Swamp	10/7/12	2/13/13	129	QS_AO_4.48.13
Q. symmetrica	KR063335	n/a	Bear Swamp	10/7/12	2/13/13	129	QS_AO_4.48.14
Q. symmetrica	KR063336	n/a	Bear Swamp	10/7/12	2/13/13	129	QS_AO_4.48.15
Q. symmetrica	KR063337	n/a	Bear Swamp	10/7/12	2/13/13	129	QS_AO_4.48.16
Q. symmetrica	KR063338	n/a	Bear Swamp	10/7/12	2/13/13	129	QS_AO_4.48.17
Q. symmetrica	KR063339	n/a	Bear Swamp	10/7/12	2/13/13	129	QS_AO_4.48.2
Q. symmetrica	KR063340	n/a	Bear Swamp	10/7/12	2/13/13	129	QS_AO_4.48.7
Q. symmetrica	KR063341	n/a	Bear Swamp	10/7/12	2/13/13	129	QS_AO_4.48.9
N. flabelullum	n/a	KP691357	Hawley Bog	4/29/13	5/3/13	4	NF_AO_4.125.7
H. elegans	n/a	KP691367	Hawley Bog	10/11/11	2/18/12	130	HE_AO113.1.1
H. elegans	n/a	KP691338	Hawley Bog	10/11/11	2/7/12	119	HE_AO_1.103.3
H. elegans	KF476109	KP691337	Hawley Bog	10/11/11	2/18/12	130	HE_AO113_2
H. elegans	n/a	KP691336	Hawley Bog	10/11/11	3/4/12	145	HE_AO122_20.1
H. elegans	n/a	KP691374	Hawley Bog	10/7/12	4/9/13	1	He_AO_4.100.17
H. elegans	n/a	KP691379	Hawley Bog	10/7/12	4/9/13	184	HE_AO_4.100.6

H. elegans	n/a	KP691370	Acadia	8/10/12	2/15/13	189	HE_AO_4.52.14
H. elegans	KF476105	KP691334	Hawley Bog	8/27/09	9/30/09	34	HE_DL3_63_7.2
H. elegans	n/a	KP691335	Hawley Bog	4/29/13	5/3/13	4	LAK63646
H. papilio	n/a	KP691371	Hawley Bog	10/7/12	2/15/13	127	HP_AG10.2
H. papilio	n/a	KP691376	Hawley Bog	3/26/12	3/28/12	2	HP_AO2.04.6
H. papilio	n/a	KP691375	Acadia	8/10/12	4/27/13	260	HP_AO_4.114.4
H. papilio	n/a	KP691333	Hawley Bog	4/29/13	4/30/13	1	HP_AO_4.117.8
H. papilio	KF476083	KP691372	Hawley Bog	10/19/10	2/4/11	108	HP_DL3_62_2.4
H. papilio	KF476074	KP691369	Hawley Bog	10/19/10	2/4/11	108	HP_DL3_62_5.1.1
H. papilio	KF476084	KP691386	Hawley Bog	10/19/10	2/4/11	108	HP_DL3_62_8.3
H. elegans	KF476066	KP691385	Hawley Bog	8/27/09	9/30/09	34	HE_DL3_62_9.4
H. elegans	KF476070	KP691384	Hawley Bog	4/7/10	4/9/10	2	HE_DL3_99_1.1
H. elegans	KF476069	KP691368	Hawley Bog	4/7/10	4/9/10	2	HE_DL3_99_3.2
H. papilio	KF476080	KP691390	Hawley Bog	4/7/10	6/9/10	63	HP_TN2_137_2.4
N. carinata	n/a	KP691347	Acadia	8/10/12	2/15/13	189	NC_AO_4.52.2
N. carinata	KF476101	KP691348	Hawley Bog	4/7/10	7/3/10	87	NC_DL3_121_1.4
N. marginata	n/a	KP691344	Hawley Bog	10/11/11	11/8/11	28	NMA_AO_1.67.2
N. marginata	n/a	KP691345	Hawley Bog	10/11/11	11/8/11	28	NMA_AO_1.67.3
N. tincta	n/a	KP691350	Acadia	8/10/12	11/6/12	88	NT_AO_2.21.12
N. tincta	n/a	KP691355	Hawley Bog	4/29/13	5/3/13	4	NT_AO_4.125.2
N. tincta	n/a	KP691354	Hawley Bog	4/29/13	5/3/13	4	NT_AO_4.125.3
N. tincta	n/a	KP691353	Hawley Bog	4/29/13	5/3/13	4	NT_AO_4.125.5
N. tincta	n/a	KP691356	Hawley Bog	4/29/13	5/3/13	4	NT_AO_4.125.6
N. tincta	n/a	KP691349	Acadia	8/10/12	2/15/13	189	NT_AO4.52.11
Q. symmetrica	KF476100	KP691363	Bear Swamp	10/11/11	12/6/11	56	QS_AO_80.1
Q. symmetrica	n/a	KP691359	Bear Swamp	10/7/12	1/5/13	90	QS_AO_4.36.5
N. tincta	n/a	KP691352	Acadia	8/10/12	9/19/12	40	NT_AO2.65.6
H. elegans	n/a	KP691332	Acadia	8/10/12	8/10/12	0	HE_ANP_3wo.4

Table S3. Inferred amino acids and nucleotides from COI alignment

**Dinucleotide in Alignment**	**Inferred Amino Acid**	**Normal Triplet**	**Inferred Missing Nucleotide**
TA	Y	TAY	TAT
TA	L	TTR	TTA
TG	L	TTR	TTG
AT	I	ATW	ATT
GC	A	GCN	GCT
CA	S	TCN	TCN OR CCN
CT	S	TCN	TCN OR CCN
