# Supplementary figures and images for: Are microbes fundamentally different than macroorganisms? Convergence and a possible case for neutral phenotypic evolution in testate amoeba (Amoebozoa: Arcellinida)
Source: R Soc Open Sci. 2015 Dec 16;2(12):150414. doi: 10.1098/rsos.150414 (PMC4807447; doi:10.1098/rsos.150414)

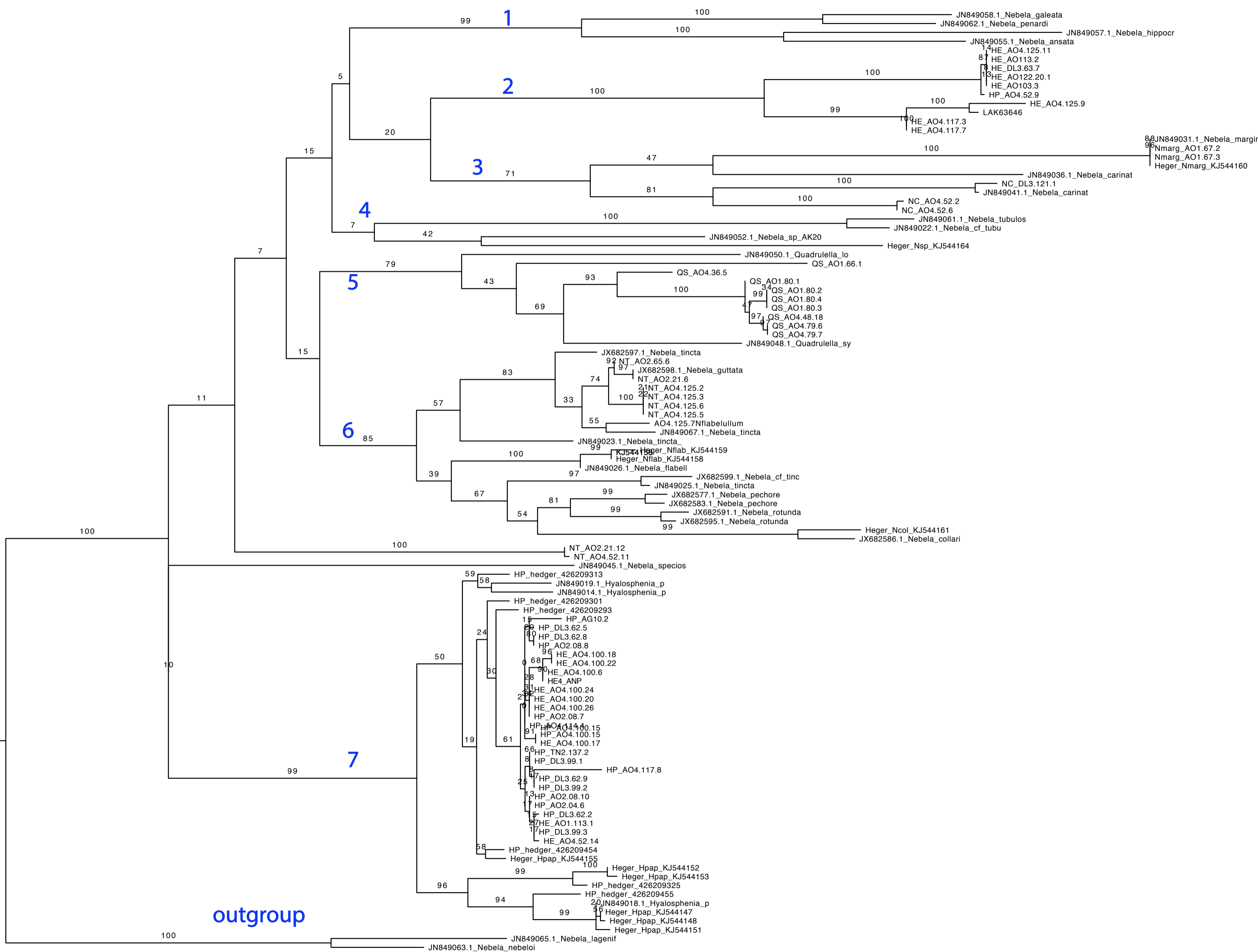

0.06

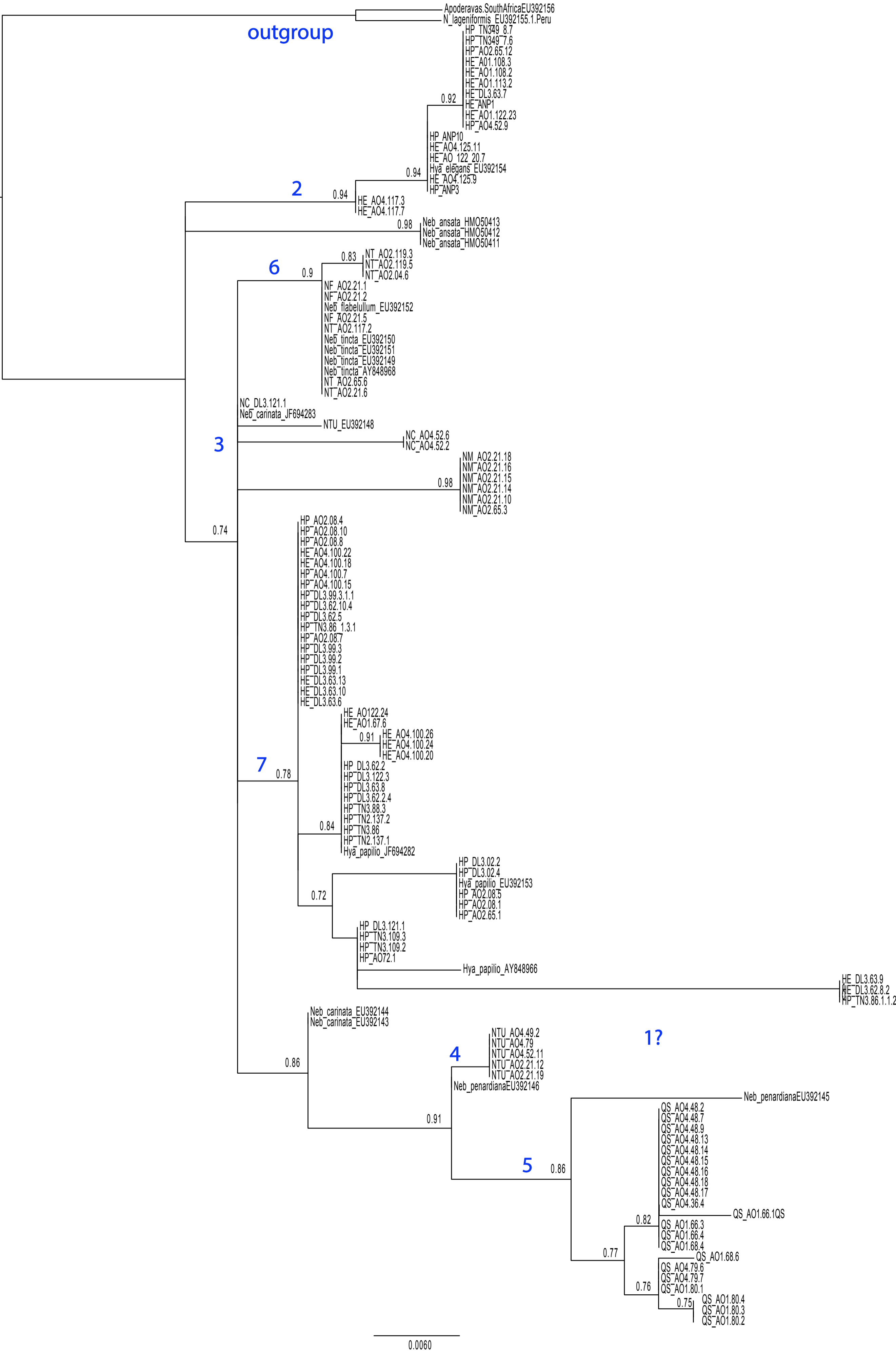

Supplement: cox1_suppfigs.pdf [file rsos150414supp2.pdf]
